# Molecular and Structural Discrimination of Proline Racemase and Hydroxyproline-2-Epimerase from Nosocomial and Bacterial Pathogens

**DOI:** 10.1371/journal.pone.0000885

**Published:** 2007-09-12

**Authors:** Maira Goytia, Nathalie Chamond, Alain Cosson, Nicolas Coatnoan, Daniel Hermant, Armand Berneman, Paola Minoprio

**Affiliations:** Laboratoire d'Immunobiologie des Infections à Trypanosoma, Département d'Immunologie, Institut Pasteur, Paris, France; Temasek Life Sciences Laboratory, Singapore

## Abstract

The first eukaryotic proline racemase (PRAC), isolated from the human Trypanosoma cruzi pathogen, is a validated therapeutic target against Chagas' disease. This essential enzyme is implicated in parasite life cycle and infectivity and its ability to trigger host B-cell nonspecific hypergammaglobulinemia contributes to parasite evasion and persistence. Using previously identified PRAC signatures and data mining we present the identification and characterization of a novel PRAC and five hydroxyproline epimerases (HyPRE) from pathogenic bacteria. Single-mutation of key HyPRE catalytic cysteine abrogates enzymatic activity supporting the presence of two reaction centers per homodimer. Furthermore, evidences are provided that *Brucella abortus* PrpA [for ‘proline racemase’ virulence factor A] and homologous proteins from two *Brucella spp* are *bona fide* HyPREs and not ‘one way’ directional PRACs as described elsewhere. Although the mechanisms of aminoacid racemization and epimerization are conserved between PRAC and HyPRE, our studies demonstrate that substrate accessibility and specificity partly rely on contraints imposed by aromatic or aliphatic residues distinctively belonging to the catalytic pockets. Analysis of PRAC and HyPRE sequences along with reaction center structural data disclose additional valuable elements for *in silico* discrimination of the enzymes. Furthermore, similarly to PRAC, the lymphocyte mitogenicity displayed by HyPREs is discussed in the context of bacterial metabolism and pathogenesis. Considering tissue specificity and tropism of infectious pathogens, it would not be surprising if upon infection PRAC and HyPRE play important roles in the regulation of the intracellular and extracellular amino acid pool profiting the microrganism with precursors and enzymatic pathways of the host.

## Introduction

In recent years, an increasing interest rose concerning Proline Racemases (PRAC). Originally isolated in 1957 from *Clostridium sticklandii* (*Cs*PRAC) [1], PRAC has been extensively studied in the eighties by several groups at the biochemical level [2], [3]. Lately, the first eukaryotic PRAC was isolated from the *Trypanosoma cruzi* pathogen (*Tc*PRAC) and shown to be involved in the mechanisms of parasite escape from host immune responses for its mitogenic properties toward B lymphocytes [4], [5]. *Tc*PRAC is present in all *T. cruzi* life cycle stages, is essential for parasite viability and it appears to be involved in certain metabolic pathways during metacyclogenesis as parasites overexpressing *TcPRAC* genes gain better host infectivity [6]. Similar genes in the human genome lack crucial enzyme catalytic residues thus consolidating *Tc*PRAC as a lead for drug development against trypanosomiasis [7], [8].

Racemases catalyze the deprotonation/reprotonation of the chiral carbon (C^α^) of both amino acid enantiomers resulting in steroinversion of chiral centers in reactions depending or not on pyridoxal phosphate (PLP) cofactor. PRAC is a member of the PLP-independent enzyme family along with Glutamate and Aspartate Racemases and Diaminopimelate Epimerase [9]. Thermodynamic studies and the overall 3D-structure of homodimeric *Tc*PRAC in complex with its competitive inhibitor provided evidences that proline (Pro) racemization operates by stabilization of carbanionic transition-state species in a two-Cystein-dependent acid/base catalytic mechanism [10]. As demonstrated by site-specific mutagenesis, racemization of Pro involves two catalytic cystein (Cys) residues (Cys_130_ and Cys_300 _) per *Tc*PRAC subunit.

Multiple alignment of functional PRAC amino acid sequences and the analysis of the conserved Cys has enabled the definition of minimal essential motifs (DRSP**C**GXGXXAXXA, i.e. MIII*, and M**C**GH) [8] to identify putative PRACs. We investigated PRAC homologous genes from pathogens by screening released genoma databases to further explore novel potential therapeutic targets. When MIII* signature was used for mining, 111 hits were obtained, 92 of them possessing both catalytic residues.

The presence of functional PRAC was investigated in a collection of 9 bacterial species of pathogenic importance (i.e. Firmicute, α-, β- and γ-proteobacteria) using molecular and biochemical approaches. Current work unveils a new functional PRAC isolated from *Clostridium difficile* and 5 novel functional Hydroxyproline-2-Epimerases (HyPRE) specifically from *Pseudomonas aeruginosa, Burkholderia pseudomallei* and 3 *Brucella* species. The studies also reveal that MIII*, considered to be a minimal pattern to identify putative PRAC, is not sufficiently stringent to discriminate PRAC from HyPRE. Additional element motifs are provided for the discrimination of PRAC and HyPRE sequences based for instance on polarity constraints imposed by precise residues of the catalytic pockets that contribute to ligand specificity.

HyPRE, a PLP-independent enzyme described in the late 1950's [11], [12], presents overall sequence similarities with PRAC but reacts only with the C^α^ of 4-hydroxyproline (OH-Pro). Enzymatic activities of bacterial PRAC and HyPREs identified here were fully characterized and specific *V_max_* and *K_m_* determined. Furthermore, the data discloses that HyPRE enzymatic activity equally depends on two catalytic Cys residues, as shown by single mutation of Cys_88_ or Cys_236_ residues of *P. aeruginosa* HyPRE which drastically impairs OH-Pro epimerization. This is the first work associating simultaneously full-length HyPRE genes and functional enzymatic activity of the encoded proteins.

The present data challenges recently published studies [13] and establishes that a *Brucella abortus* virulence factor (PrpA), described as PRAC, as well as homologous proteins from *B. melitensis* and *B. suis,* are *bona fide* PLP-independent HyPREs that interconvert *trans* or *cis* OH-L-Pro into *cis* or *trans* OH-D-Pro respectively and no other amino acids.

Both Pro and OH-Pro are important compounds for growth and development of many organisms. They can be used as exclusive sources of carbon, nitrogen and energy and are the principal components of collagen-the most widespread molecule in higher organisms [14], [15]. Moreover, the importance of PRAC and HyPRE in the context of disease processes induced by pathogenic microorganisms is discussed.

## Results

### In silico gene selection of homologous PRAC genes

Blast searches of NCBI and Swiss-Prot/TrEMBL databases with full-length *TcPRAC* sequences resulted in 184 hits from which 111 possess the minimal PRAC stringent MIII* among which 62 hits were directly annotated as ‘PRAC’, without previous validation of the enzymatic activity. The present analysis revealed that MIII* and M**C**GH motif [10], encompassing the *Tc*PRAC Cys_300_ and Cys_130_ crucial residues respectively, were consistently present in 92 sequences. We formerly suggested that predicted proteins originated from genes lacking these key Cys residues would display functions other than Pro racemization [8]. A collection of 15 sequences was selected for further studies accordingly to sequence identities with *Tc*PRAC, to the conservation or not of homologous Cys_130_ and Cys_300 _and the recognized pathogenic importance of the microbial genomes.

As summarized in Table 1, homologous genes from different pathogen strains, annotated as ‘putative PRAC’, ‘PRAC’ or ‘unknown’ proteins, display 29 to 56% homology with *Tc*PRAC, present either a conservation of the couple of catalytic Cys or replacements of one or both Cys positions by serine (Ser) and/or threonine (Thr) residues. A comparison between *Brucella spp* sequences and the previously characterized *Tc*PRAC and *Cs*PRAC was of note. Therefore, from the two available homologous sequences for each *Brucella* specie only one meets the requirements for PRAC activity and presents both key Cys residues, the other presenting Ser and Thr substitutions.

**Table 1:** Database collection from in silico searches and complementary information on selected sequences.

Pathogen	Disease	Acces. nb†	MCGH§	MIII* §	*Tc*PRAC Homology (%)	Annot.‡
*Trypanosoma cruzi* CL Brener	Chagas' disease	Q868H8	C	C	100	PRAC
*Bacillus anthracis* Ames	Anthrax	Q81PH1	C	C	40	Put. PRAC
		Q81UH1	C	C	40	
*Brucella abortus* 9-941	Brucellosis	Q57B94 (1)	C	C	40	Put. PRAC
		Q57F22 (2)	S	T	29	
*Brucella melitensis* 16M	Brucellosis	Q8YJ29	C	C	40	PRAC
		Q8YFD6	S	T	29	
*Brucella suis* 1330	Brucellosis	Q8FYS0	C	C	40	Put. PRAC
		Q8G2I3	S	T	29	
*Burkholderia cenocepacia* HI2424	Pneumonia, sepsis	A0AZQ0	C	C	29	PRAC
		A0B0B8	C	T	37	
*Burkholderia pseudomallei* K96243	Melioidosis	Q63NG7	C	C	34	Hyp. prot
*Clostridium difficile* 630	Nosocomial diarrhoea	Q17ZY4	C	C	56	Put. PRAC
*Pseudomonas aeruginosa* PAO1	Pneumonia, sepsis	Q9I476	C	C	33	Hyp. prot
		Q9I489	S	C	30	
*Vibrio parahaemolyticus* O3 :K6	Diarrhoea	Q87Q20	C	C	37	PRAC

Sequences were obtained by blasting *Tc*PRAC (Q868H8) against Swiss-Prot/*Tr*EMBL or NCBI databases.

† Swiss-Prot accession number;

§ MCGH and MIII

* motifs are minimal peptide sequences encompassing *Tc*PRAC catalytic Cys (Cys_130 _and Cys_300_) residues;

‡ Related annotation from blast searches.

### Functional PRAC and Hydroxyproline epimerases of pathogenic bacteria

**Figure 1:**
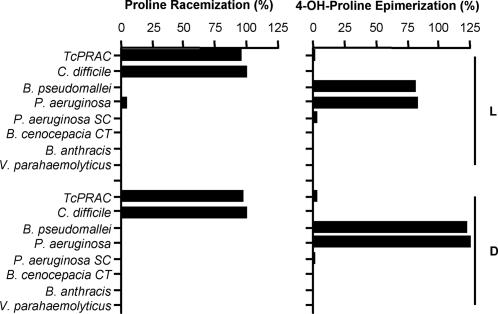
Enzymatic activities of PRAC and HyPRE from different pathogens. Optimal reaction conditions consisted of 10 µg of the enzyme and 20 mM of substrate in specific buffers during 30 min at 37°C. (A) Percent of L- or D-Pro racemization in NaOAc, pH 6; (B) Percent of OH-L-Pro or OH-D-Pro epimerization in TE, pH 8. *P. aeruginosa* (SC) and *B. cenocepacia* (CT) recombinant proteins whose sequences lack one of the two Cys catalytic residues do not display any PRAC or HyPRE activities.

The function of 12 gene products and their ability to interconvert Pro residues was addressed. Purified recombinant proteins were analyzed in biochemical assays by measuring the shift in optical rotation of either L- or D-Pro. As shown in Figure 1, *C. difficile (Cd)* recombinant protein racemized both L- and D-Pro but not OH-L/D-Pro or any other natural amino acid. *Cd*PRAC activity is PLP-independent which closely resembles *Tc*PRAC and *Cs*PRAC [5], [16]. Conversely, *B. pseudomallei* and *P. aeruginosa* recombinant proteins presented no measurable PRAC activity but demonstrated strong epimerization of OH-L/D-Pro behaving as genuine OH-Pro epimerases. However, as predicted, control recombinant proteins produced from *B. cenocepacia* and *P. aeruginosa* sequences that present ‘Cys-Thr’ or ‘Ser-Cys’ couple replacements respectively did not show neither PRAC nor HyPRE enzymatic activities. Unexpectedly, three tested recombinant proteins, two produced from *Bacillus anthracis* and one from *Vibrio parahaemolyticus*, annotated as ‘putative PRACs’ and presenting the ‘Cys-Cys’ couple generated recombinant proteins that did not display PRAC or HyPRE activities.

### Brucella abortus PrpA virulence factor is a validated hydroxyproline epimerase

**Figure 2:**
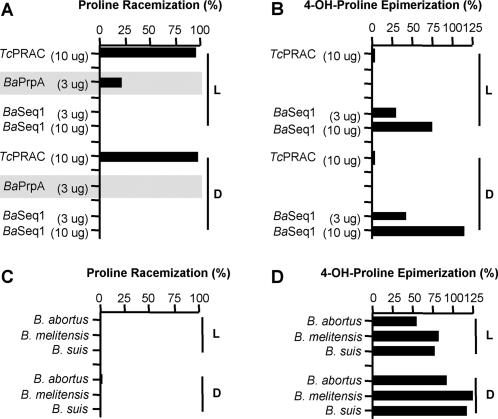
PrpA of B. abortus (BaSeq1) is an hydroxyproline-2-epimerase. Reactions were performed with 3–10 µg of the enzyme and 40 mM of substrate in specific buffers during 1 h at 37°C. (A) Pro racemization reactions were performed in NaOAc, pH 6. (B) OH-Pro epimerization reactions were set up in parallel in TE, pH 9. Data from Spera et al [13] was transposed to the Figure under shade and *Tc*PRAC was used as control; *Ba*PrpA : purported ‘proline racemase protein A’; *Ba*Seq1 was produced from PrpA-corresponding sequence 1 from *B. abortus* (Table 1 and Figure S1). Percent of L- or D-Pro racemization (C) and percent of OH-L-Pro or OH-D-Pro epimerization (D) using specific buffers, 10 µg of the enzyme and 20 mM of substrate during 30 min at 37°C.

One of the *B. abortus* sequences, presenting the ‘Cys-Cys’ couple, was reported elsewhere as a B-cell mitogen with PRAC activity (*Ba*PrpA, for proline racemase protein A) and was shown to be directly involved in bacterial virulence and immune system evasion [13]. Surprisingly, PrpA would not be able to perform reversible conversion of L- and D-Pro but only a L-Pro to D-Pro unidirectional conversion. If correct, this assertion would imply that other PRAC could behave likewise. The enzymatic activity of *Ba*PrpA produced from sequence 1 obtained *in silico* was then investigated. *Ba*Seq1, derived from *Ba*-strain 544, is 100% homologous to *Ba*-strain 9-941 and *Ba*PrpA (*Ba* strain 2308, BAB1_1800) and possesses all PRAC motifs (Figure S1). The present data undoubtedly demonstrate that *Ba*Seq1 displayed only HyPRE activity irrespective of the enzyme concentration, pH and buffer conditions (Figures 2A and 2B), in contrast to recurrent Pro racemization values obtained with *Tc*PRAC. Since *Ba*Seq1 sequence is 98% homologous to proteins annotated as ‘PRAC’ from *B. melitensis (Bm)* and *B. suis (Bs)* all three recombinant homologous proteins were tested in parallel for PRAC and HyPRE activities. These proteins were unable to catalyze Pro racemization but exhibited equivalent strong ability to perform epimerization of both OH-L-Pro and OH-D-Pro (Figure 2 C and 2D). Consequently, the data proves that *Ba*Seq1, and therefore PrpA, is a HyPRE, as do *B. melitensis* and *B. suis* corresponding proteins.

### Kinetic properties of bacterial hydroxyproline epimerases

**Figure 3:**
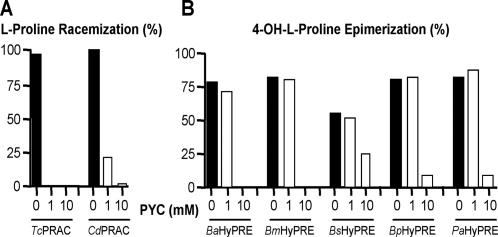
Pyrrole-2-carboxylic acid (PYC), the specific inhibitor of PRAC, is not an inhibitor of HyPRE. (A) Percent of L-Pro racemization or (B) OH-L-Pro epimerization in absence (black bars) or in presence of 1 or 10 mM of PYC (white bars). Reactions were performed at 37°C for 30 min with 10 µg of the corresponding enzymes in NaOAc, pH 6 (PRAC reactions) or TE, pH 8 (HyPRE reactions) and 20 mM of substrate.

**Figure 4:**
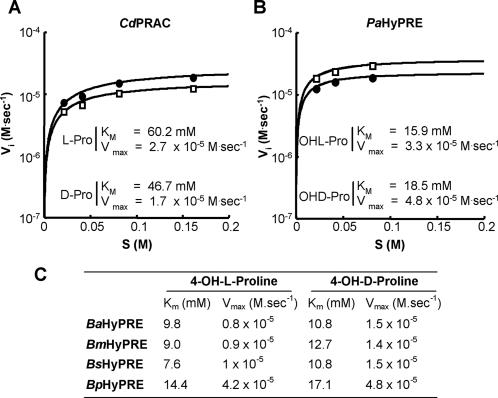
Kinetic parameters of Proline racemization and Hydroxyproline epimerization. Progress of enzymatic activities was monitored polarimetrically, as described previously [8]. Initial rates were plotted in function of [S] and kinetic parameters determined with Kaleidagraph® program and Michaelis-Menten equation. Maximum rate (*V_max_*) and Michaelis-Menten constant (*K_m_*) were obtained at 37°C by incubation of 20 µg/ml of each recombinant protein with increasing concentrations of specific L- (closed circles) or D- (open squares) substrates. (A) PRAC activity is depicted for *C. difficile*; (B) HyPRE activity is depicted for *P. aeruginosa*. (C) *K_m_* and *V_max_* records of HyPRE reactions using L- or D- enantiomers were distinctively obtained with recombinant enzymes of *B. abortus* (*Ba*HyPRE), *B. melitensis* (*Bm*HyPRE), *B. suis* (*Bs*HyPRE) and *B. pseudomallei* (*Bp*HyPRE). *Tc*PRACA : *K_m_* of 29 mM *and V_max_* of 5,3×10^−5 ^M.sec^−1^ and TcPRACB : *K_m_* of 75 mM and *V_max_* of 2×10^−4^ M.sec^−1^.

Optimum conditions for PRAC and HyPRE reactions for all bacterial enzymes were obtained in NaOAc, pH 6 and Tris/EDTA (TE), pH 8–9 buffers, respectively. On the other hand, when PRAC was radically inhibited by its specific competitive inhibitor pyrrole-2-carboxylic acid (PYC), no inhibition of HyPRE was observed with standard amounts of PYC (1 mM) (Figures 3A and 3B). HyPRE reactions were only affected by high amounts of PYC (10 mM) or by variable concentrations of iodoacetate and iodoacetamide inhibitors (Figure S2). Progress of Pro and OH-Pro catalysis was monitored polarimetrically. The interconversion of L<>D-Pro mediated by *Cd*PRAC reveals that the enzyme has comparable velocity and affinity constants to those of *Tc*PRAC (Figure 4). Graphic representation of the Michaelis-Menten equation corresponding to the initial velocity of *Cd*PRAC and *Pa*HyPRE as function of substrate concentration is shown, as well as respective *K_m_* and *V_max_* kinetic values (Figure 4A and 4B). *Brucella spp* and *B. peudomallei* HyPREs exhibited comparable *V_max_* and apparent *K_m_* values to those of *Pa*HyPRE (Figure 4C). However, at equilibrium, all HyPRE enzymes showed a clear advantage to OH-D-Pro substrate.

### Additional elements to previously defined PRAC signature can discriminate proline racemases and hydroxyproline epimerases

**Figure 5:**
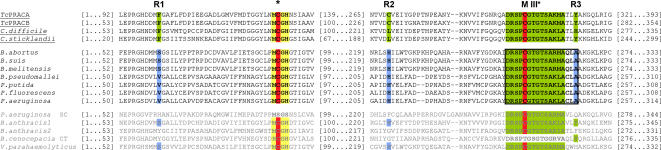
Alignments of PRAC and HyPRE protein sequences. MCGH and MIII* PRAC motifs are shaded respectively in yellow and green. Catalytic Cys residues are coloured in red. R1, R2 and R3 indicate critical compulsory differences allowing for the discrimination of PRAC and HyPRE. In the left margin, sequences corresponding to PRAC are underlined contrasting to HyPRE sequences (plain text). Residues involved in substrate specificity are shaded in green (PRAC) or blue (HyPRE). The proposed signature for HyPRE (squared) gather an additional block of specific residues downstream PRAC MIII*. Sequences that do not meet those requirements and thus present unknown functions are in light gray.

Following blast searches using full-length *Tc*PRAC sequence, MIII* and MCGH block, we demonstrated that a number of homologous hits corresponded to HyPRE, a PRAC-related enzyme. Sequences of PRAC and HyPRE were aligned and residues that may be useful for their discrimination were identified (Figure 5). Thus, although both enzymes possess the catalytic ‘Cys-Cys’ couple, three major and non dissociated differences seem to be noteworthy for substrate specificity. The first and most important particularity is an aromatic phenylalanine (Phe) residue which was shown to be capital to hydrophobic contacts of *Tc*PRAC with Pro ring carbon atoms that is missing in HyPRE (depicted in R1). In fact, Phe imposes polarity constraints precluding polar functions at the level of the substrate carbon ring. Instead, HyPRE holds Ser or valine (Val) substitutions, i.e. small polar or aliphatic amino acids, that would account for better OH-Pro accessibility into the pocket. Other sequences encoding proteins without enzymatic activity may present at that position, polar tyrosine (Tyr) or histidine (His) residues which would restrict PRAC or HyPRE catalysis, as observed with *B. anthracis* sequences. Another feature is the presence in the *Tc*PRAC pocket environment of a Cys (or a Leucine, i.e. Leu, for other PRAC) residue in position 270 while HyPREs possess in that position a consistent polar His residue (depicted in R2) optimally placed to favor H-bonding interaction with the OH- of the C^γ^-atom of OH-Pro. Moreover, an additional block of three residues downstream of the highly conserved MIII* (XLA, depicted in R3) is fully restrictive to discriminate HyPRE and PRAC enzymes. These three differences are complementary to the presence of the ‘Cys-Cys’ couple of the catalytic pockets as ascertained by the absence of both enzymatic activities exhibited by *B. anthracis* and *V. parahaemolyticus* proteins.

### Abrogation of HyPRE enzymatic activity by mutation of conserved cysteine residues of the catalytic site

**Figure 6:**
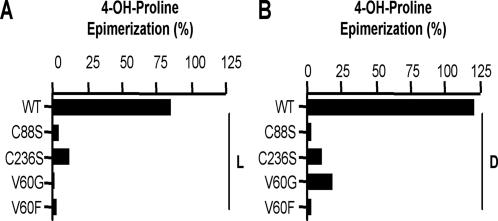
Site-directed mutagenesis of key residues of PaHyPRE results in loss of enzymatic activity. (A) and (B) Percent of epimerization of respectively OH-L-Pro and OH-D-Pro exhibited by WT *Pa*HyPRE or C88S, C236S, V60G and V60F point mutants.

HyPRE homodimer was described as having both subunits participating in a single catalytic site [17], [18]. The potential role of ‘Cys-Cys’ couple in HyPRE catalysis was verified through site-directed mutagenesis of *Pa*HyPRE Cys_88_ or Cys_236_ into Ser residues (Figure S3 and Table S1). In comparison to wild type HyPRE, ^C88S^HyPRE and ^C236S^HyPRE single mutations induce radical loss of OH-L/D-Pro epimerization establishing that proton transfer during HyPRE reaction is indeed dependent on the presence of the catalytic ‘Cys-Cys’ couple of each subunit (Figure 6). To validate the weight of the Val_60_ residue in ligand accessibility and thus in substrate specificity, the residue was mutated into glycine (Gly) (^V60G^HyPRE) or Phe (^V60F^HyPRE), meeting or not size and stability limits imposed by Val. The absence of epimerization exhibited by the two mutants revealed that the Val_60_ aliphatic residue indeed accounts for OH-Pro ligand specificity and is consequently essential for HyPRE catalysis. Conversely, the Phe_102_ residue on the PRAC catalytic site environment offers hydrophobic restriction area to the pocket occupancy restraining the accessibility of OH-Pro.

**Figure 7:**
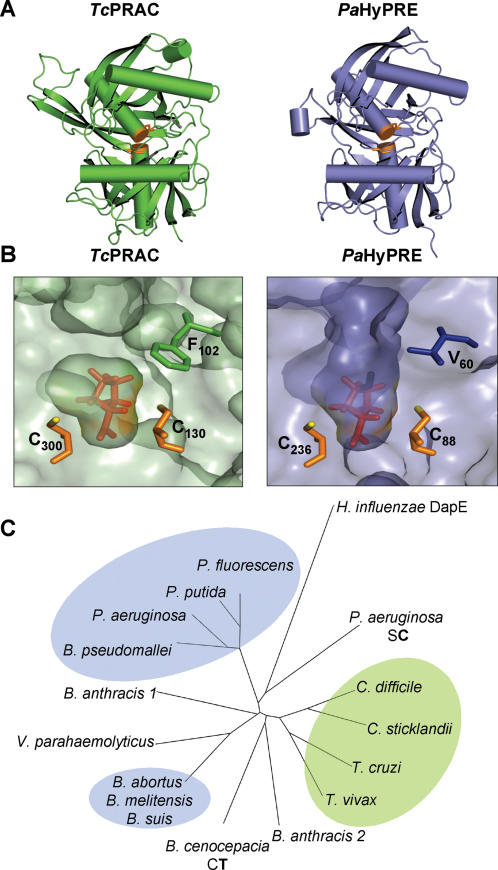
PRAC and HyPRE structural data, pocket constraints and evolution. (A) Ribbon model of *Tc*PRAC (green, PDB : 1W61) and *Pa*HyPRE (purple, PDB : 2AZP) subunits revealing the overall similarities of the 3D-structures. Cys catalytic residues (orange). (B) Close view of *Tc*PRAC (left panel) and *Pa*HyPRE (right panel) pockets. The two catalytic Cys residues of PRAC (C_130_ and C_300_) and of HyPRE (C_88_ and C_236_) are shown in the reaction center colored in orange sticks. Hydrophobic F_102_ (green sticks) and aliphatic V_60_ (blue sticks) residues are depicted respectively in PRAC and HyPRE reaction centers where Pro and OH-Pro were modeled. Polarity hindrance imposed by the aromatic PRAC F_102_ residue and the solvent accessible area for the ligand made possible by HyPRE V_60_ residue are shown. (C) Phylogram of PRAC and HyPRE aligned sequences showing the unrooted tree using *H. influenzae* DapE as uncontroversial outgroup. Bacterial and protozoa PRAC cluster together suggesting that divergence of PRAC and HyPRE took place before the separation of bacteria and eukaryotes.

The space and polarity constraints of PRAC and HyPRE active sites on protein–ligand interactions are visualized better by comparing the closer views of the enzyme pockets (Figure 7A and 7B). Therefore, despite close similarities displayed by PRAC and HyPRE 3D-structures, the presence of a sizable aromatic residue or, alternatively, of a small aliphatic or polar amino acid, unquestionably plays a determinant role on the enzyme/substrate specificity.

The significance and conservation of PRAC and HyPRE throughout evolution was investigated by a phylogram using another PLP-independent enzyme as an uncontroversial outgroup, i.e. the *Haemophilus influenzae* diaminopimelate epimerase (DapE). Figure 7C shows that PRAC and HyPRE cluster in three main groups. Interestingly, PRAC from *C. difficile* and *C. sticklandii* cluster together with *T. cruzi* and *T. vivax* (*Trypanosoma vivax* possesses a functional proline racemase. N. Chamond, A. Cosson, M. Goytia, P. Minoprio, 2007, manuscript to be submitted), the segregation of the tree branches reflecting their ancient origin. It is conceivable that the divergence between PRAC and HyPRE is phylogenetically older than the separation of bacteria, archea and eukaryotes. Alternatively, possible gene transfer between species can be envisaged.

## Discussion

The discovery of novel microbial genes and metabolic proteins through genome mining has proven to be a promising approach to identify potential candidates for drug discovery and therapy against infections. Despite increased availability of genome data, the attribution of putative functions to homologous genes annotations are at times too simple and errors can occur with the consequence of incorrect scientific dogmas. In this paper we report that from a selected database assembled from blast searches using *T. cruzi* proline racemase (*Tc*PRAC) full-length sequences, 73% of the hits were incorrectly annotated as PRAC or putative PRAC since most of the proteins do not experimentally display functional PRAC activity. Consistent with previous data determining critical residues for PRAC catalysis, the present study reveals that out of 12 ‘PRAC-like’ recombinant proteins from different pathogens only one (8%), from *C. difficile,* which is a significant nosocomial pathogen [19], [20], demonstrates truthful PRAC activity. Other proteins isolated from bacteria responsible for human and animal health problems [21]–[25] have been incorrectly annotated as PRAC and are in fact HyPRE, i.e. 3 *Brucella* species, *P. aeruginosa* and *B. pseudomallei*. In addition, 33% of the studied sequences were erroneously annotated though missing fundamental catalytic residues. To our knowledge, apart from previous work using purified *P. putida* HyPRE which associated the enzyme active site to 14 residues [18], the current work is the first describing HyPRE full-length genes and may contribute in the future to better annotation of unknown ORFs.

Based on overall comparisons between PRAC and HyPRE and despite the evident identities displayed by the peptide sequences, structural evidences were presented here that allow the discrimination of both enzymatic activities. Considering the results obtained with *Pa*HyPRE mutants supporting the key role of Cys_88_ and Cys_236_ residues in catalysis and the large overall structural similarity with *Tc*PRAC, our data supports a reaction mechanism similar to PRAC where HyPRE equally possesses two active sites per dimer, each one including two catalytic Cys. Therefore, Cys_88_ and Cys_236_ residues are correctly positioned in the HyPRE pocket to perform epimerization of C^α^ OH-Pro chiral center. However, HyPRE is not inhibited by PYC, the transition state analogue of Pro. It has previously been shown that hydrophobic Phe_102_ (R1) and Phe_290 _residues present in the *Tc*PRAC pocket impose polarity restrictions that enable interactions of the enzyme with the C^α^ Pro ring or the C2 atom of PYC. Instead, the absence of Phe residues in HyPRE pocket, most particularly Phe_102_, and its substitution by an aliphatic Val (or polar Ser), promotes an ideal environment for accessibility and stereoinversion of the C^α^ of OH-Pro. Indeed, mutagenesis of Val_60_ into Gly or Phe, results in radical loss of *Pa*HyPRE activity, attributing a significant role to Val_60_ in the conformation of the enzyme, the pocket stability and the ligand specificity. It could though be hypothesized that a single replacement of this central Val_60_ residue by an aromatic Phe would be sufficient to affect the hydrosolubility of the HyPRE pocket environment thus favouring the accessibility and correct positioning of ‘Pro’ and its further ‘racemization’. Nonetheless, our data shows that this hypothesis is unlike since *Pa*HyPRE V60F-mutant is unable to perform L-Pro < > D-Pro conversion. *Per se* this result is not surprising given that racemization of Pro by PRAC catalytic Cys is known to be assisted by neighbor residues of the pocket that are equally present on HyPRE, such as Leu_127_, His_132_ and Asp_296_, [10], but in addition by Phe_290_ which is absent in *Pa*HyPRE sequence. These neighboring residues may be involved in significant hydrophobic interactions of the enzyme with its ligand and influence the *pKa* of the catalytic Cys residues thus determining the environment hydrophobicity and as such affecting the stability of the pocket and resulting catalysis.

On the other hand, PRAC and HyPRE multiple alignments allowed identification of other important and non dissociated elements that account for the discrimination of the enzymes, such as the presence of the aliphatic Cys (or Leu) residue in *Tc*PRAC at position 270 which is absent and replaced by a polar His residue in HyPRE (R2) thus favouring its interaction with OH-Pro. Additionally, a block of residues (XLA) downstream of the previously identified minimal MIII* PRAC signature [7], [8] was found to be HyPRE-specific (R3). The combination of those elements are fundamental in shaping the binding pocket and thus determining the substrate specificity as supported by the detailed structural analysis of *Tc*PRAC and *Pa*HyPRE active sites. It would be interesting to verify if multiple replacements of discriminating R1, R2 and R3 HyPRE elements by PRAC specific residues would induce any changes in substrate specificity. Nevertheless, although HyPRE and PRAC catalytic sites are structurally very similar, full-sequence disparities between these two enzymes are still substantial (app. 35% homology). Consequently, conformational factors (non-R1, non-R2, non-R3) that contribute to substrate recognition are certainly more subtle and intricate to distinguish than R1, R2 and R3 whose identification was exclusively based on sequence ‘(dis)similarities’. Eventhough, several coexistent mutations might introduce drastic reductions in *k_cat_* and account for a restrained catalytic activity of the mutant and/or “asymmetric” preferences for a particular substrate stereoisomer, thus affecting “racemic principles”. It is also conceivable that major (extra) distortions in the pocket geometry and charge delocalization-previously shown to play a role on ligand accessibility [10]–may occur thus interfering with the analysis.

We report here a clarification of earlier work [13] concerning an immunomodulatory virulence factor (PrpA) of *B. abortus*, that possibly due to its 40% homology with *Tc*PRAC was described as a PRAC. Surprisingly, PrpA was described as displaying discrete racemization of L-Pro but as unable of catalyzing the conversion of D-Pro enantiomer. As such, this data would imply that some racemases do not follow fundamental racemic principles. The present data establishes that PrpA from *B. abortus, B. melitensis* and *B. suis* are in fact HyPRE that catalyze the interconversion of OH-L/D-Pro. These results are significant to prevent any misinterpretations of mechanisms linked to pathogenesis induced by *Brucella spp*.

HyPRE is a PLP-independent enzyme, shown to be essential in *P. putida* that, like other *Pseudomonas spp,* has been found to cause nosocomial infections with resulting septicemia and septic arthritis [22], [24], [26]. Here we identify functional HyPRE from *P. aeruginosa* and several other important pathogens such as *B. pseudomallei* and *Brucella spp* agents of melioidosis and brucellosis, respectively [21], [23], [25]. These human and animal pathogens affect multiple systems and result in abcesses, pneumonia and fatal septicemia in immunosuppressed hosts. Bacterial meningitis can also provoke collagen degradation and break-down of blood-brain barrier, which consequently raises bacterial invasiveness and persistence resulting in brain injuries [27]. Interestingly, OH-L-Pro and L-Pro, are the major constituents of collagen, the main component of the extra-cellular matrix, making up 25% of the total body protein content [15]. Bacteria and viruses, deprived of collagen, have virulence factors which destroy collagen or interfere with its production by the secretion of collagenase and/or elastase [28], [29]. *P. aeruginosa*, for instance, induces disruption of blood vessels through elastase by dissolution of the elastic lamina of arteries and arterioles, or by degrading major fragments of collagen IV [30]. It is worth noting that OH-L-Pro upregulates expression of bacterial genes whose products are involved in vital metabolic pathways, such as OH-D-Pro oxidase, deaminase and dehydrogenase [31]. Specific membrane transporters for OH-L/D-Pro have been shown to exist in bacteria to increase the fundamental intracellular OH-Pro pool [32]. However, mutants lacking HyPRE are unable to metabolize OH-L-Pro and hence are not viable in OH-L-Pro containing media as the sole carbon source [31]. This fact alone confers an essential importance to HyPRE that by converting OH-L-Pro to OH-D-Pro allows its intracellular utilization by the action of OH-D-Pro oxidases. We therefore hypothesize that HyPRE, as PRAC, could serve as a good target for the development of therapies.

Finally, PRAC enzymes, as other B-cell mitogens, have been described as involved in evasion mechanisms of parasite and bacterial species through the induction of non-specific hypergammaglobulinemia and by the secretion of pleiotropic cytokines [4], [5]. We have shown that similarly to *Tc*PRAC, PRAC from *C. difficile* and HyPREs from *P. aeruginosa* and *B. abortus* are also strong lymphocyte mitogens as they increase *in vitro* lymphoproliferation by up to 10 fold (Table S2). It has been shown that mitogen-induced proliferation of resting lymphocyte is associated with a marked increase in amino acid uptake and intracellular enzyme pathways to meet the demands of increased cellular protein synthesis [33]. It is relevant that enzymes of Pro biosynthesis, and not those of Pro degradation, are particularly increased with lymphocyte activation. However, with sufficient amounts of exogenous Pro, large increases are observed of pyrroline-5-carboxylate reductase (PCA reductase), a key enzyme in Pro synthesis. Isoforms of PCA reductase, sensitive and insensitive to feedback inhibition by Pro do exist [34]. Interestingly, PCA reductase from distinct tissues differs according to its sensitivity to Pro-inhibition. Considering tissue specificity and tropism of infectious pathogens, it would not be surprising if upon infection PRAC and HyPRE play important roles in the regulation of the intracellular and extracellular amino acid pool profiting the microrganism with precursors and enzymatic pathways of the host.

## Materials and Methods

### Data mining and bioinformatics

*Tc*PRAC sequence (AF195522, NCBI, E.C.5.1.1.4) and PRAC motif III* were used to blast genome databases. Default settings for Blast were used. Unrooted trees and alignments were obtained with ClustalW program.

### Bacterial strains and DNA extraction

Purified DNA was obtained from *B. anthracis* (strain 9131), *C. difficile* (strain VPI10463), *V. parahaemolyticus* (CNRVC 010089), *B. abortus* (strain 544), *B. melitensis* (strain 16M), *B. suis* (strain 1330) and *B. pseudomallei* (strain K96243). DNA was extracted from bacterial pellets of *B. cenocepacia* (strain J2315) and *P. aeruginosa* (strain PAK) with the DNA tissue culture extraction kit (Qiagen)

### Primer design, gene cloning and recombinant Proteins

Forward and Reverse primers were designed based on *Tc*PRAC sequence toward specific sequences of the genes of interest (Table S2, Supplemental files). Bacterial PCR products were purified by QuickPCR Qiaprep kit (Qiagen) and cloned into BamHI/EcoRI or BamHI/NcoI sites of pET28b (Novagen/Merck) using Rapid Ligation Kit (Roche). *E. coli* DH5α cells were transformed with empty or ligated plasmids. Plasmids were extracted with the Qiaprep Spin Miniprep kit (Qiagen) from bacterial pellets from individual colony cultures and sequenced (Genome Express, Meylan/France). Sequences, ORFs and the presence of C-terminal 6x-His Tag were verified. *E. coli* BL21 (DE3) cells were transformed with ligated plasmids. Recombinant proteins were purified as described [7], [8].

### Enzymatic activity assays

Optimum racemization and epimerization conditions were determined using 20 mM L-Pro or OH-L-Pro in 0.2 M NaOAc or Tris 20mM/EDTA 1 mM (TE) buffers respectively, as a function of pH. Percent of racemization or epimerization of serial concentrations of substrate was calculated by incubating 3–10 µg of recombinant protein, 20–80 mM substrate in NaOAc pH 6 or TE, pH 8 (q.s.p. 500 µl) for 30–60 min at 37°C. The reactions were stopped by incubating at −20°C and optical rotations measured in a polarimeter 241MC (Perkin Elmer) [8]. Percent inhibition of enzymatic activities was determined incubating 10 µg of recombinant protein in presence or absence of 1–10 mM PYC, 1–25 mM iodoacetamide, or 1–25 mM iodoacetate. Control reactions were performed in presence or absence of PLP. All reagents were purchased from Sigma.

### Kinetic assays

Assays were performed at 37°C with 10–160 mM of each substrate, 20 µg/ml of specific enzymes in optimum reaction buffer [8]. Briefly, after determination of the linear part of the curve, velocity in 10–160 mM substrate was measured every 30 s during 5 min to determine *K_m_* and *V_max_*.

### Site directed mutagenesis

Site-directed mutagenesis of *Pa*HyPRE was performed using a QuikChange XL kit (Stratagene), as described [10], to obtain the point mutants C88S, C236S, V60F and V60G. Briefly, point mutations were obtained by PCR using forward and reverse overlapping mutagenic primers (Figure S3 and Table S2). Plasmids were purified and point mutations were ascertained by sequencing. Recombinant proteins were produced from each point mutant, as described above.

### Accession Numbers

The following nucleotide sequences were submitted to GenBank™ Data Bank with accession numbers EF495346 (*Cd*PRAC, *C. difficile* VPI10463), EF495341 (*Pa*HyPRE, *P. aeruginosa* PAK), EF495342 (*Bm*HyPRE, *B. melitensis* 16M), EF495343 (*Bs*HyPRE, *B. suis* 1330), EF495344 (*Ba*HyPRE, *B. abortus* 544), EF495345 (*Bp*HyPRE, *B. pseudomallei* K96243).

## Supporting Information

Figure S1 Sequence alignments of Brucella abortus PrpA and PrpB virulence factors.
(1.27 MB TIF)

Figure S2 Inhibition of HyPRE reactions with alkylating agents.
(0.29 MB TIF)

Figure S3 Strategy for PaHyPRE site specific mutagenesis.
(0.33 MB TIF)

Table S1 Primers used for the production of recombinant proteins and site-directed mutagenesis.
(0.42 MB TIF)

Table S2 Mitogenic activity of PRAC and HyPRE enzymes.
(0.40 MB TIF)
