# Supplementary figures and images for: Molecular and Structural Discrimination of Proline Racemase and Hydroxyproline-2-Epimerase from Nosocomial and Bacterial Pathogens
Source: PLoS One. 2007 Sep 12;2(9):e885. doi: 10.1371/journal.pone.0000885 (PMC1964878; doi:10.1371/journal.pone.0000885)

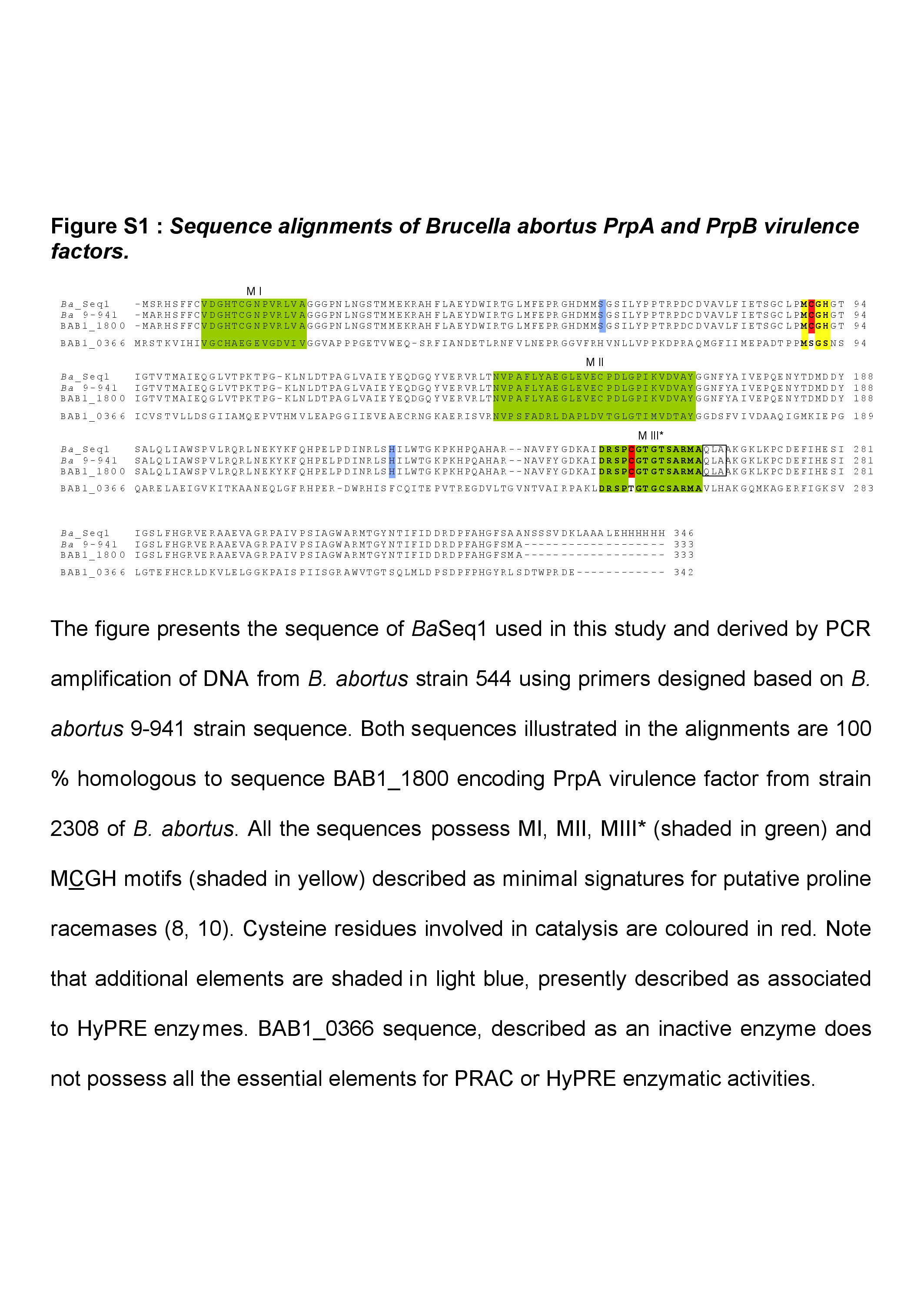

Supplement: Figure S1 — Sequence alignments of Brucella abortus PrpA and PrpB virulence factors. (1.27 MB TIF) [file pone.0000885.s001.tif]

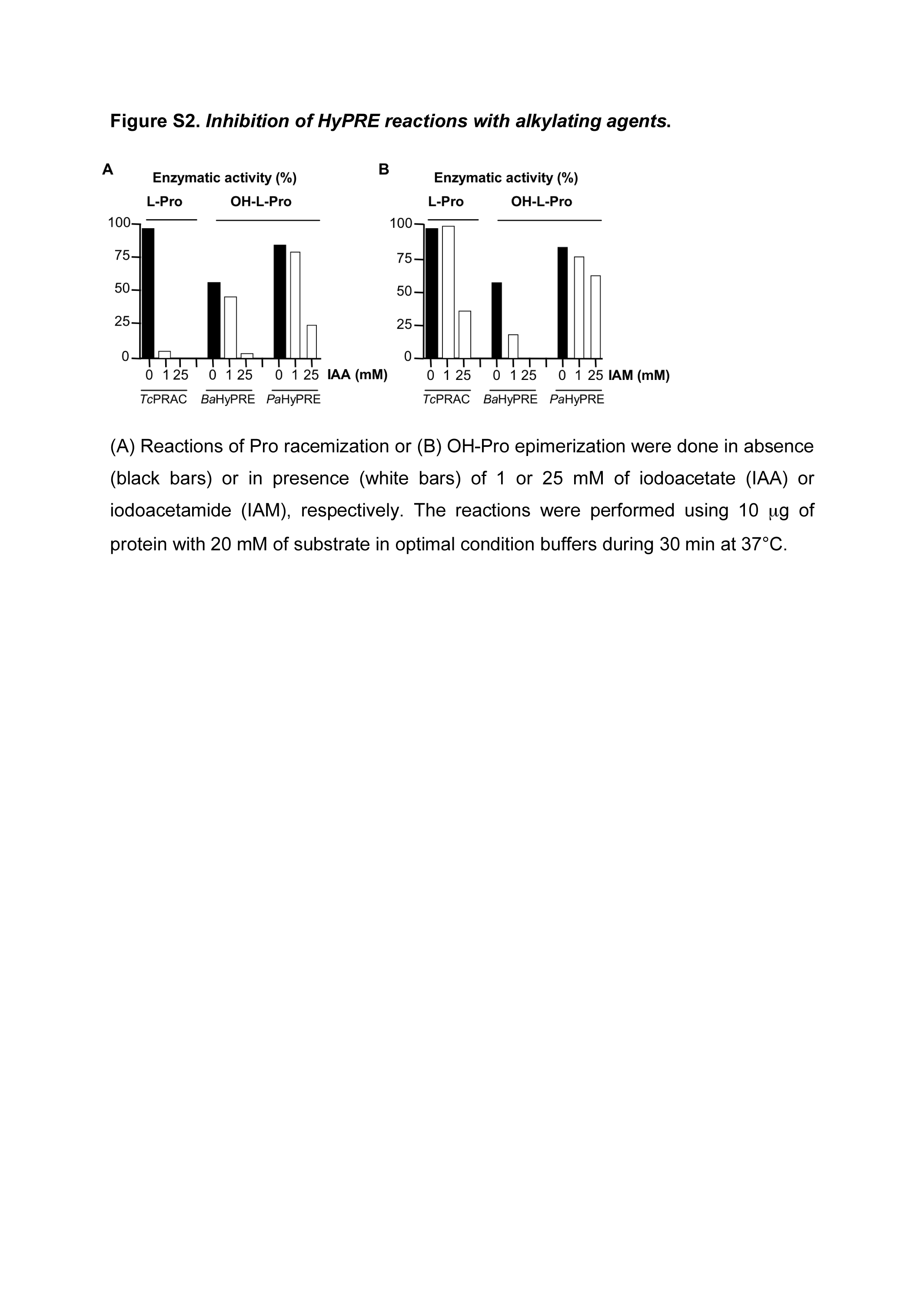

Supplement: Figure S2 — Inhibition of HyPRE reactions with alkylating agents. (0.29 MB TIF) [file pone.0000885.s002.tif]

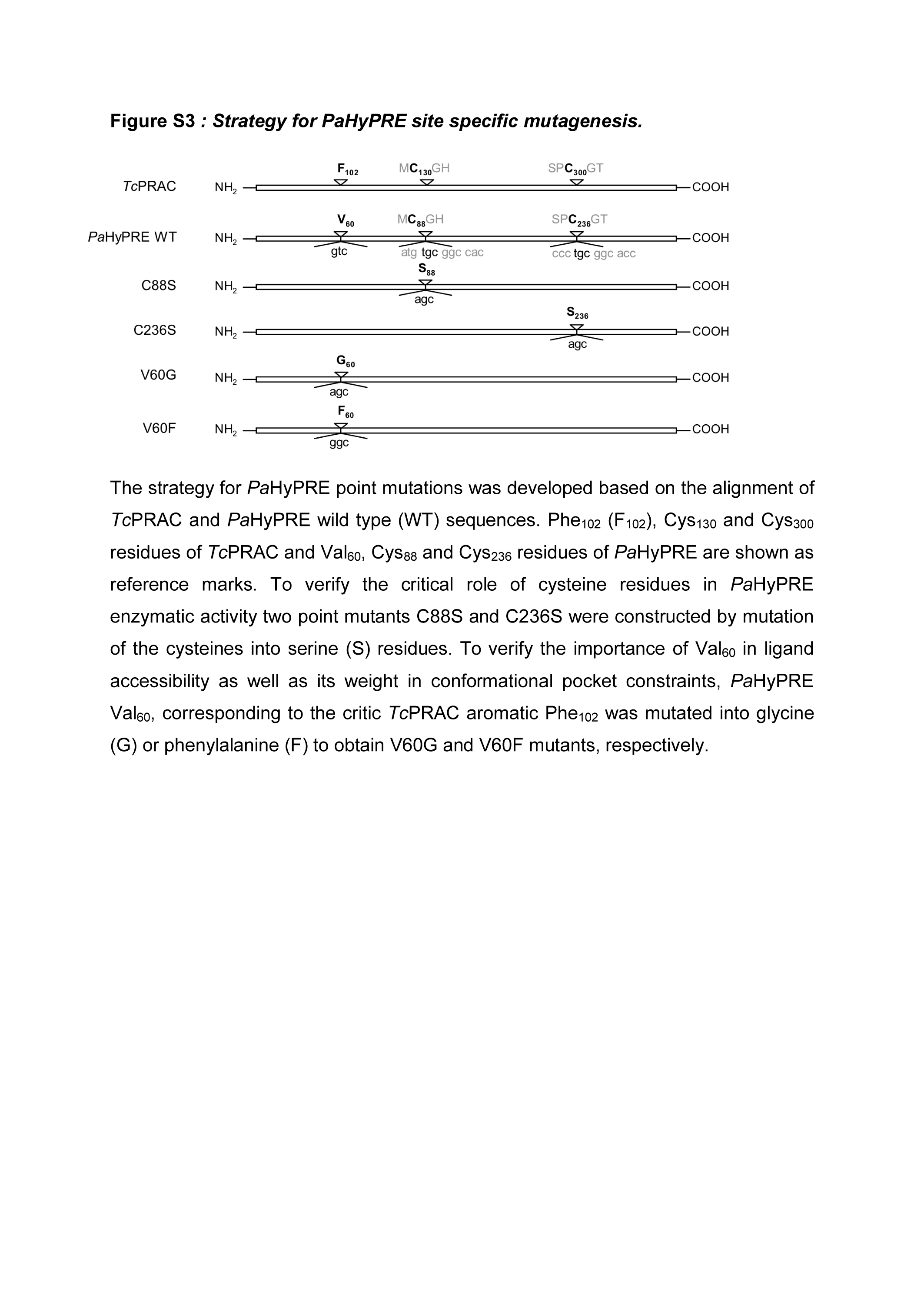

Supplement: Figure S3 — Strategy for PaHyPRE site specific mutagenesis. (0.33 MB TIF) [file pone.0000885.s003.tif]

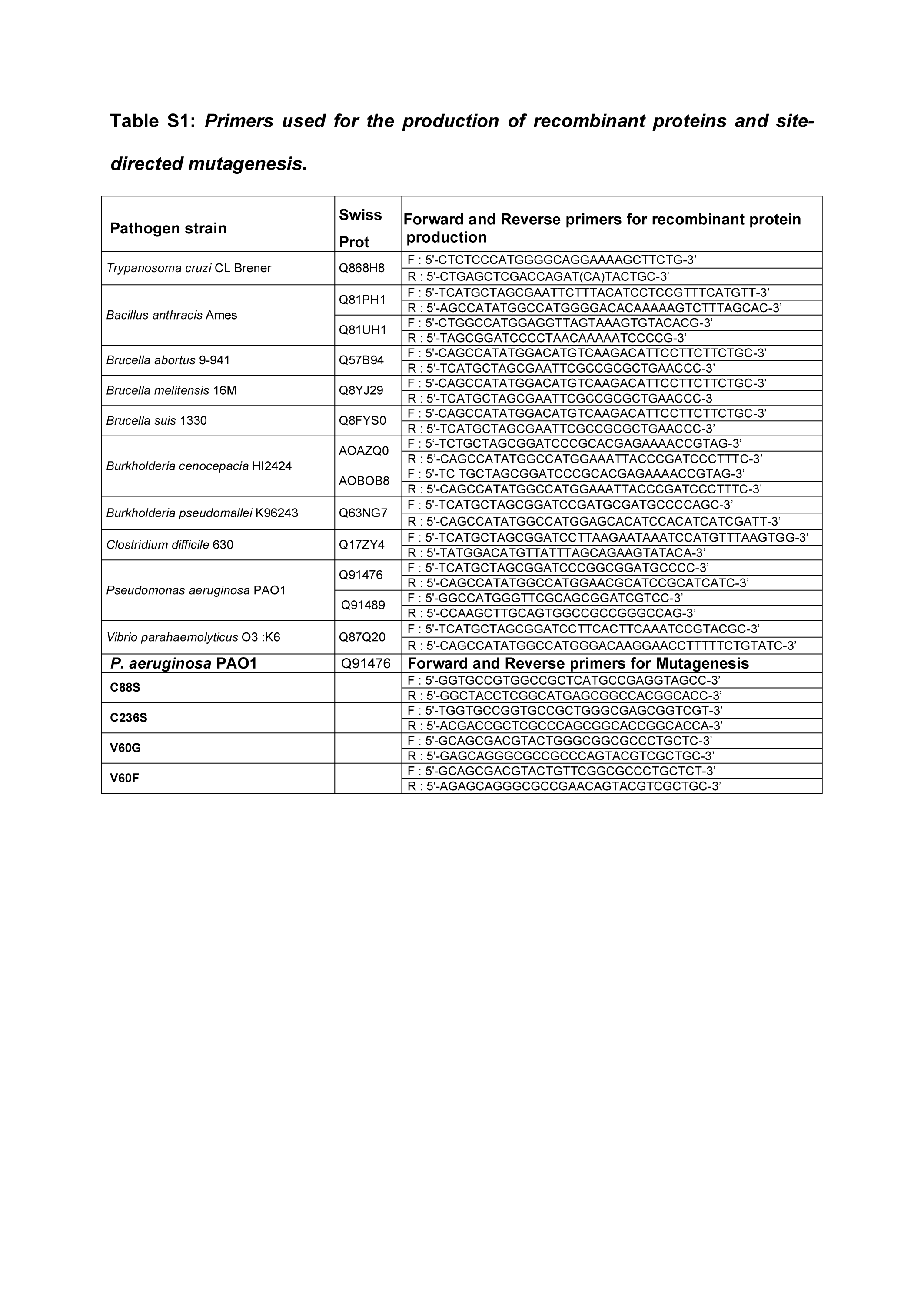

Supplement: Table S1 — Primers used for the production of recombinant proteins and site-directed mutagenesis. (0.42 MB TIF) [file pone.0000885.s004.tif]

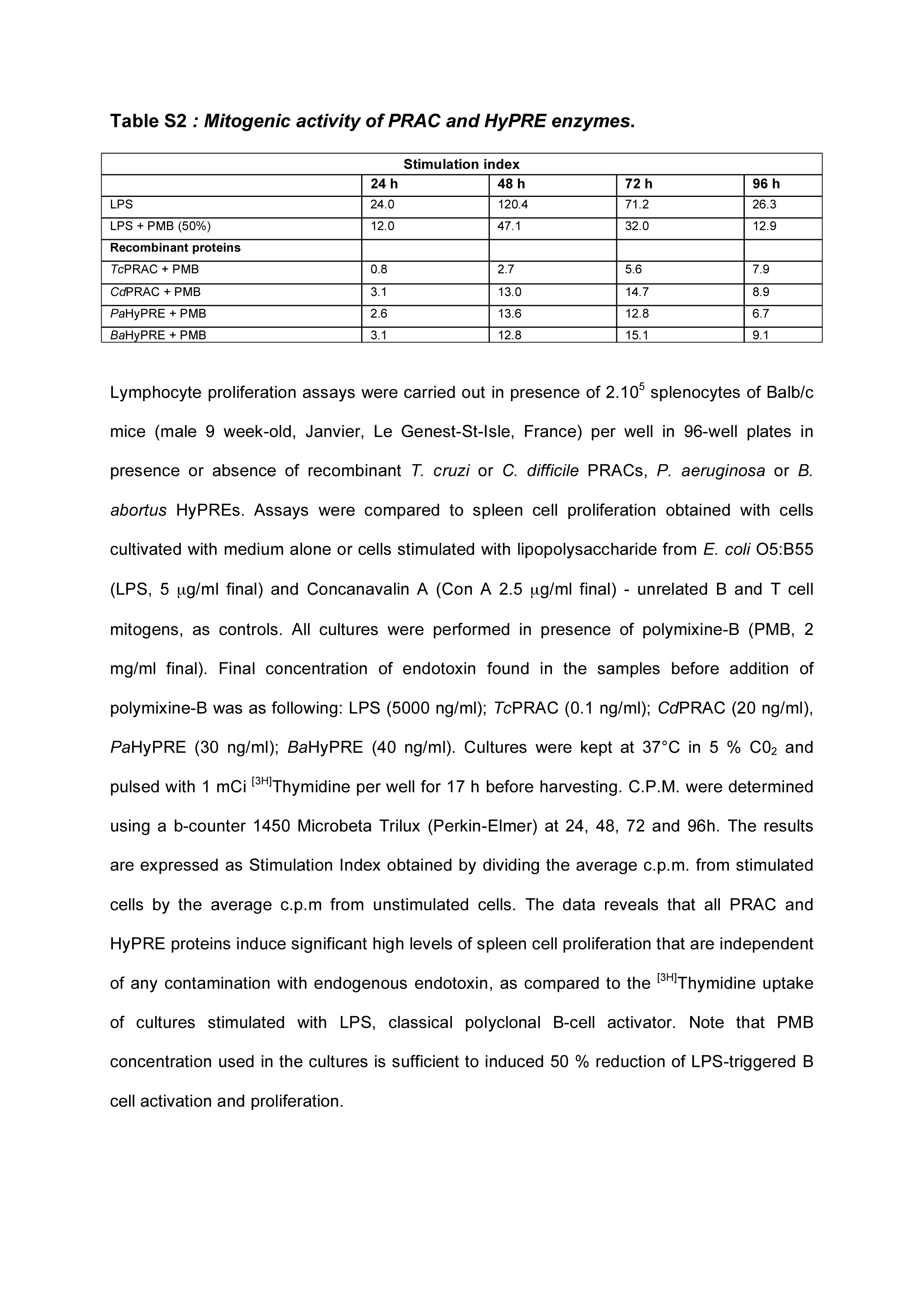

Supplement: Table S2 — Mitogenic activity of PRAC and HyPRE enzymes. (0.40 MB TIF) [file pone.0000885.s005.tif]
